# Acute effects of foam rolling on jump performance and neuromuscular responses following resistance exercise

**DOI:** 10.3389/fphys.2026.1945152

**Published:** 2026-09-09

**Authors:** Coşkun Rodoplu, Josef Fischer, Mert Arabacı, Yahya Yıldırım, Hüseyin Topçu, Ufuk Şekir, Andreas Konrad

**Affiliations:** 1 Department of Common Courses, Rectorate, Bursa Technical University, Bursa, Türkiye; 2 Institute of Human Movement Science, Sport and Health, Graz University, Graz, Austria; 3 Department of Physical Education and Sports Teaching, Faculty of Sport Sciences, Bursa Uludağ University, Bursa, Türkiye; 4 Department of Sport Medicine, Faculty of Medicine, Bursa Uludağ University, Bursa, Türkiye

**Keywords:** horizontal jump, neuromuscular fatigue, self-myofascial release, surface electromyography, vertical jump

## Abstract

**Introduction:**

A single bout of high-load resistance exercise can induce acute neuromuscular fatigue, necessitating effective recovery strategies to maintain explosive performance. However, the effects of foam rolling (FR) on vertical and horizontal jump performance and muscle excitation following fatiguing resistance exercise remain unclear. This study examined the acute effects of FR compared with passive recovery (PR) on vertical and horizontal jump performance and lower-extremity muscle excitation following a fatiguing resistance exercise protocol.

**Methods:**

Thirty-one healthy, physically active men (age: 21.6 ± 1.3 years; body mass: 75.3 ± 10.3 kg; height: 180.6 ± 7.2 cm) completed a randomized crossover trial comparing FR and PR. Measurements were obtained at pre-test, immediately after exercise (mid-test), and after an 8-min recovery intervention (post-test). Jump performance was assessed using the countermovement jump (CMJ) and single-leg hop for distance (SLHD), while surface electromyography was recorded from the quadriceps, hamstrings, and gastrocnemius muscles during both tasks.

**Results:**

A significant condition × time interaction was observed for CMJ and SLHD performance (p<.05; $\eta_{\text{p}}^{2}=.216-.256$). Compared with PR, the FR condition showed improvements in CMJ (Δ = 6.23%; p = .004) and SLHD performance (Δ = 10.27%; p<.001) from mid-test to post-test. No significant condition × time interaction was found for normalized muscle excitation (p >.05; $\eta_{\text{p}}^{2}=.002-.070$), although time-dependent changes were observed only in the rectus femoris and gastrocnemius muscles during the SLHD test (p<.05; $\eta_{\text{p}}^{2}=.187-.251$).

**Conclusion:**

These findings indicate that FR facilitated the post-exercise restoration of vertical jump performance and produced an acute enhancement in horizontal jump performance following resistance exercise, yet the performance benefits were not accompanied by measurable changes in normalized muscle excitation. The results underscore the potential of FR as a practical recovery tool for improving functional performance, while the underlying physiological mechanisms remain uncertain.

## 1 Introduction

Resistance exercises play a fundamental role in improving muscular strength, power, and functional capacity and is considered a key component in enhancing athletic performance while reducing injury risk (Fragala et al., 2019; Varela-Olalla et al., 2020). A single session of high-load resistance exercise (e.g., 80% one-repetition maximum [1RM] half squat) can elicit substantial neuromuscular stimulation while simultaneously inducing measurable acute fatigue (Sun et al., 2024; Rodoplu et al., 2026b). These acute responses may result in transient alterations in physical performance outcomes and neuromuscular function (Fonseca et al., 2020; Kotikangas et al., 2024). Therefore, the implementation of appropriate post-exercise recovery strategies is important for regulating the acute neuromuscular load and preserving physical performance following resistance exercise (Fischer et al., 2025; Petrigna et al., 2025).

Surface electromyography (sEMG) is widely used to assess neuromuscular responses by providing direct information regarding the timing and magnitude of muscle excitation (Davis et al., 2025). The single-leg hop for distance (SLHD) and countermovement jump (CMJ) tests are commonly employed to evaluate horizontal and vertical jump performance, respectively, while the concurrent assessment of sEMG during these tasks enables the characterization of activation patterns in the quadriceps, hamstrings, and gastrocnemius muscles, as well as the identification of potential neuromuscular compensation strategies (Bhanot et al., 2019; García-Arrabé et al., 2025). A single session of high-load half squat exercise can impose considerable neuromuscular stress and localized muscle fatigue, which may transiently alter both CMJ and SLHD performance. Therefore, integrating performance assessments with sEMG measurements may provide a more comprehensive understanding of the neuromuscular responses to resistance exercise and the recovery process following acute fatigue (Ramirez-Campillo et al., 2021; Gamage et al., 2025).

Passive recovery (PR), stretching, hydrotherapy, and massage are among the most commonly used strategies to promote post-exercise recovery (Pablos et al., 2020; Konrad et al., 2022). In recent years, foam rolling (FR) has gained considerable attention owing to its ease of application, low cost, and suitability for field-based settings (Cheatham et al., 2015; Rodoplu et al., 2025). The proposed mechanisms underlying FR include alterations in the viscoelastic properties of soft tissues, increased local blood circulation, modulation of nociceptive input, and reorganization of proprioceptive and mechanoreceptive afferent signaling (Nakamura et al., 2021; Kasahara et al., 2022). These mechanical and sensory effects may influence neuromuscular responses during subsequent movements (Behm and Wilke, 2019). Previous studies have examined muscle excitation following FR, including the hamstrings, although the findings have been inconsistent (Madoni et al., 2018; Santana et al., 2022). Previous evidence suggests that a single post-exercise FR session may facilitate recovery without adversely affecting muscle performance (20,23).

However, the evidence regarding its effects on jump performance following fatiguing exercise remains inconsistent. While some studies have reported beneficial effects on vertical jump performance after post-exercise FR, others have demonstrated trivial or even detrimental effects (Laffaye et al., 2019; Sedano and Maroto-Izquierdo, 2025; Rodoplu et al., 2026b). These inconsistent findings may be partly attributable to differences in FR protocols, the characteristics of the preceding fatiguing exercise, and the performance outcomes assessed across studies (Rodoplu et al., 2026b; Rodoplu et al., 2026a). In contrast, evidence regarding horizontal jumping performance, particularly the SLHD, post-exercise FR remains limited (Nygaard Falch et al., 2020; Rodoplu et al., 2026a). Therefore, there is a need to simultaneously examine performance outcomes and sEMG responses to determine the acute effects of post-exercise FR applied following a single session of high-load resistance exercise on vertical and horizontal jump performance, as well as the neuromuscular responses of the lower-extremity muscles, including the quadriceps, hamstrings, and gastrocnemius.

Although FR has been investigated following different exercise protocols, evidence regarding its effects following high-load resistance exercise remains limited (Laffaye et al., 2019; Sedano and Maroto-Izquierdo, 2025). Our previous studies examined balance, jump performance, and muscle excitation following explosive and complex contrast exercise (Rodoplu et al., 2026b; Rodoplu et al., 2026a). The present study extends this work by examining the effects of FR following high-load resistance exercise, with a specific focus on vertical and horizontal jump performance. Therefore, the aim of the present study was to investigate the acute effects of FR, compared with PR, on vertical jump performance assessed by the CMJ, horizontal jump performance assessed by SLHD, and the neuromuscular responses of the lower-extremity muscles (quadriceps, hamstrings, and gastrocnemius) following a single session of high-load resistance exercise. It was hypothesized that, compared with PR, FR would attenuate the acute decline in jump performance induced by resistance exercise and facilitate a faster normalization of sEMG-assessed muscle excitation.

## 2 Materials and methods

### 2.1 Participants

A total of 31 healthy, physically active male participants (age: 21.6 ± 1.3 years; body mass: 75.3 ± 10.3 kg; height: 180.6 ± 7.2 cm) volunteered for this study. Physical activity status was assessed using the International Physical Activity Questionnaire (IPAQ), with participants reporting a mean physical activity level of 1156.1 ± 808.9 MET-min/week. The required sample size was estimated using G*Power software (version 3.1.9.7; Düsseldorf, Germany) based on data reported in a similar study by Heinke et al. (2025). *A priori* power analysis was performed using an effect size of f = 0.25, an alpha level of 0.05, and a statistical power of 0.80. The analysis indicated that a minimum of 24 participants would be sufficient for a crossover study design. The study was conducted in accordance with the principles of the Declaration of Helsinki and received approval from the Clinical Research Ethics Committee of Bursa Uludağ University Faculty of Medicine (Date: 26/01/2026; Approval Code: 2026/48/2-22). The inclusion criteria were: (a) being healthy according to the Exercise Readiness Questionnaire (EGZ-A+), (b) being between 18 and 30 years of age; and (c) having no prior experience with FR. The exclusion criteria were: (a) the presence of any neurological or musculoskeletal disorder; (b) the use of stimulant substances; (c) the use of medication that could affect physical performance prior to testing; and (d) any injury within the previous three months that could influence muscle performance. Written informed consent was obtained from all participants prior to participation.

### 2.2 Experimental design

**Figure 1:**
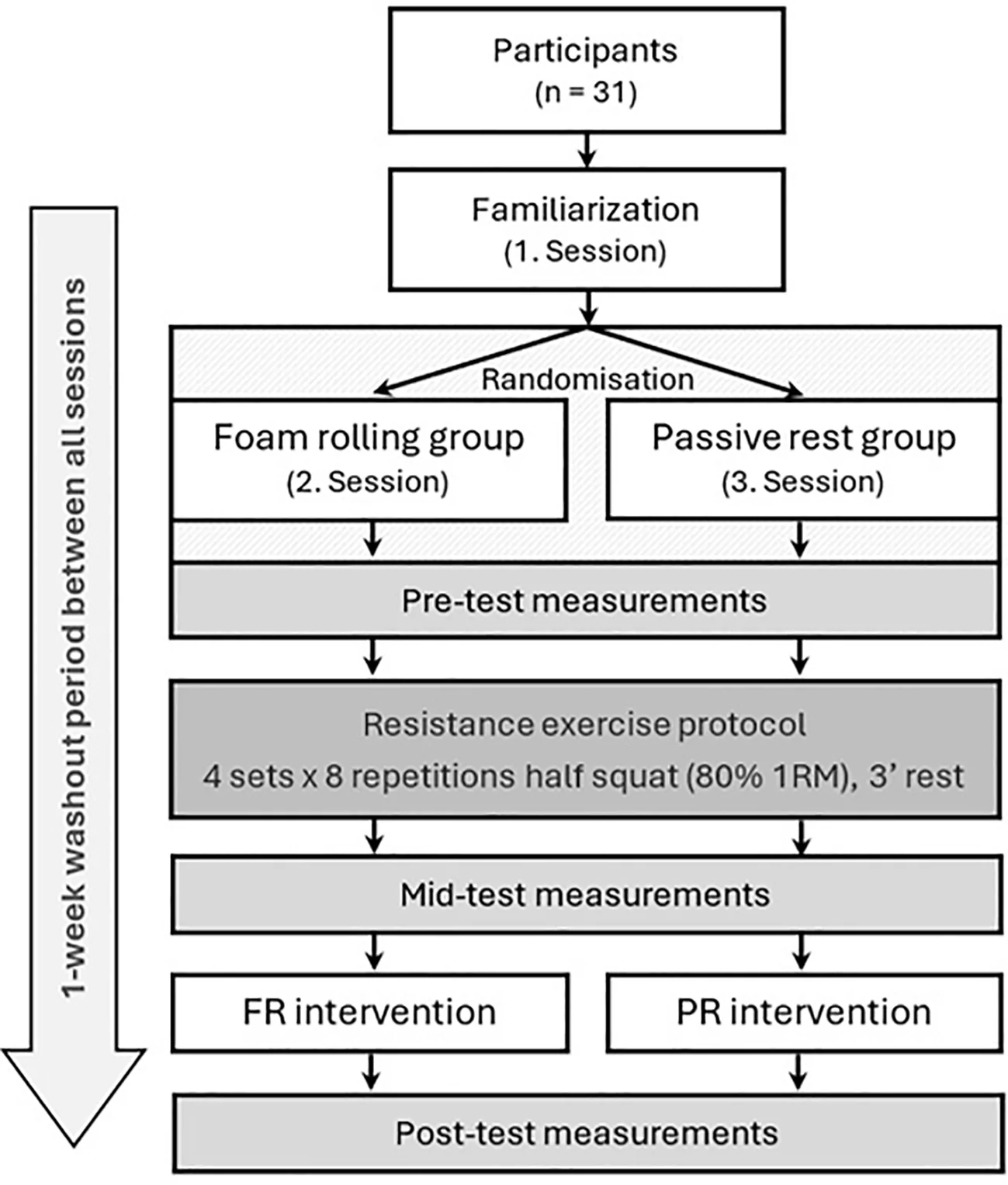
The flowchart of the study.

The study was designed using a randomized crossover design. Each participant attended the laboratory on three separate occasions: one familiarization session and two experimental sessions, with a washout period of at least one week between sessions. During the familiarization session, participants were informed about the study procedures and provided written informed consent, and the order of the FR and PR interventions was determined using an online randomization program (www.randomizer.org). The allocation sequence was concealed until participant assignment. Due to the nature of the interventions, assessor blinding was not feasible. Eligibility was assessed using the Exercise Participation Questionnaire (EGZ-A+) and the IPAQ. Body height, body mass, and body mass index were subsequently measured. To determine the exercise intensity, a 10-repetition maximum (10RM) half-squat test was performed, and individual 1RM values were estimated. Participants were then familiarized with the SLHD, CMJ, resistance exercise protocol, and FR intervention. During the experimental sessions, participants first completed a standardized warm-up consisting of 5 min of treadmill running at 6 km·h⁻¹ followed by 5 min of dynamic stretching. Following the warm-up, maximal voluntary isometric contraction (MVIC) measurements were obtained for sEMG normalization. Participants then completed the pre-test measurement, performed the half-squat exercise protocol at 80% of their estimated 1RM, completed the mid-test measurement, underwent either the FR or PR intervention, and finally completed the post-test measurement. Ratings of perceived exertion during the half-squat exercise were monitored using the Borg 6–20 Rating of Perceived Exertion (RPE) scale. The term “test” refers to the simultaneous measurement of SLHD and CMJ performance together with synchronized sEMG recordings. Participants were instructed to refrain from strenuous exercise for 48 h before each experimental session. All experimental sessions were performed at the same time of day under controlled laboratory conditions (20–22 °C). The study flowchart is presented in **Figure 1**.

### 2.3 Procedures

#### 2.3.1 Tests

Countermovement jump (CMJ): Vertical jump performance was assessed using the Sport Expert™ MPS-501 system (Tümer Elektronik LTD., Türkiye), with a measurement resolution of 0.1 cm. Jump height was automatically calculated from flight time. Participants were instructed to perform a maximal vertical jump from a semi-squat position with approximately 90° of knee flexion while keeping their hands on their hips and without using an arm swing. Three trials were performed, with a 15-s rest interval between trials. The mean jump height (cm) across the three trials was used for statistical analysis (Rodoplu et al., 2026a).

Single-Leg Hop for Distance (SLHD): Horizontal jump performance was evaluated using the SLHD. Participants were instructed to stand barefoot behind a designated starting line with their hands on their hips and perform a maximal forward hop using their dominant leg while maintaining a stable landing. Hop distance was measured using a tape measure fixed to the floor. Three trials were completed, and the mean hop distance (cm) was used for statistical analysis (Sawle et al., 2017).

#### 2.3.2 One-repetition maximum estimation

To estimate 1RM, a half squat protocol was performed in accordance with the procedures described by Smilios et al. (2005). During the familiarization session, participants first completed a general warm-up lasting approximately 10 min. Subsequently, a 10-repetition maximum (10RM) test was conducted using a standardized Smith machine. The test was performed at a half squat depth corresponding to approximately 90° of knee flexion. Squat depth was monitored using a manual goniometer and an adjustable tripod bar. Participants were instructed to lightly touch the bar with their hips during the descending phase of the movement. Based on observations made during the warm-up and participant feedback, an estimated 10RM load was selected, and participants continued the exercise until momentary muscular fatigue was reached. Movement tempo was standardized using a metronome, with each repetition lasting 4 s (2 s eccentric and 2 s concentric). Individual 1RM values were subsequently estimated from the 10RM results using the equation proposed by Brzycki (1993).

#### 2.3.3 Exercise protocol

The resistance exercise protocol consisted of 4 sets of 8 repetitions of the half squat exercise and required approximately 15 min to complete (Sun et al., 2024). A 3-min rest interval was provided between sets. This recovery period was selected to minimize the detrimental effects of fatigue on exercise performance while allowing sufficient recovery between sets and reducing the potential risk of injury (Sedano and Maroto-Izquierdo, 2025; Faigenbaum et al., 2006).

#### 2.3.4 Recovery

The recovery protocol consisted of two experimental conditions: PR and FR. During the PR condition, participants remained seated on a chair in a relaxed position for 8 min. During the FR condition, self-myofascial release was performed using a cylindrical foam roller (Blackroll, Bottighofen, Switzerland) on the hamstrings, quadriceps, and gastrocnemius muscles. All FR sessions were supervised by the same investigator. The intervention was initiated on the dominant limb and subsequently repeated on the non-dominant limb in the same sequence. For each muscle group, FR was performed for two sets of 30 s (total duration: 60 s). One complete rolling cycle (from distal to proximal and back) was completed in approximately 2 s. During FR of the hamstrings and quadriceps, participants were instructed to maintain the applied pressure at approximately 7/10 on a 10-point visual analogue scale (VAS). When the perceived pressure fell below this level, the investigator applied additional downward pressure manually (Behm et al., 2020). FR of the gastrocnemius muscles was performed using body weight only. The total duration of the FR intervention for both limbs was approximately 6 min. As the intervention required direct contact with the skin, the sEMG electrodes were temporarily removed before FR and repositioned afterward on the pre-marked anatomical locations by the same investigator. Including the transition periods between muscle groups and electrode repositioning, the overall recovery period lasted approximately 8 min.

#### 2.3.5 Surface electromyographic and normalization

**Figure 2:**
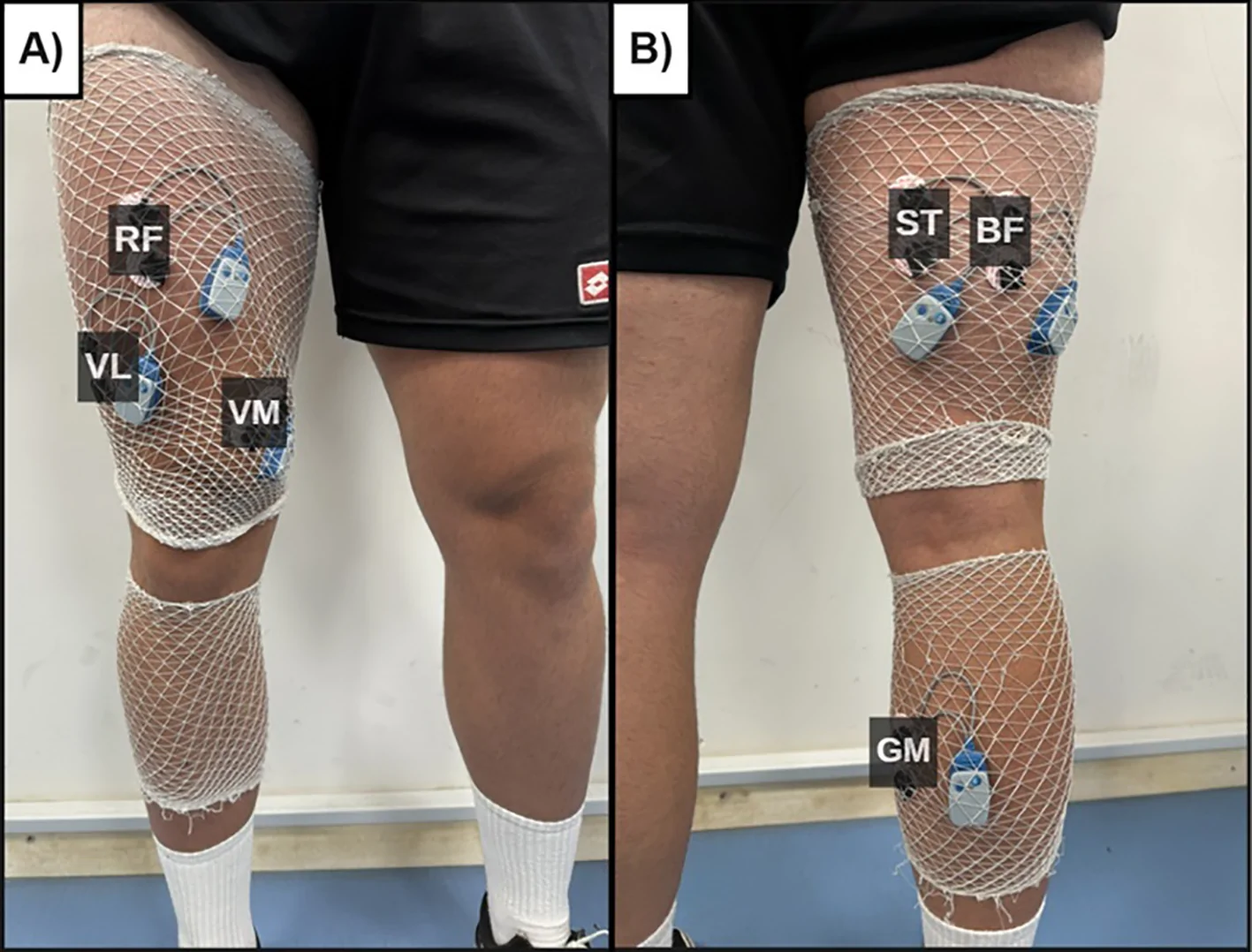
Surface electromyography electrode placement on the lower-extremity muscles: **(A)** quadriceps (rectus femoris, vastus medialis, and vastus lateralis); **(B)** hamstrings (biceps femoris and semitendinosus); and gastrocnemius.

sEMG excitation was recorded from the dominant lower limb using a portable 16-channel sEMG system (Ultium EMG, Noraxon USA Inc., Scottsdale, AZ, USA). Differential bipolar Ag/AgCl surface electrodes were placed parallel to the muscle fiber orientation over the quadriceps (rectus femoris, vastus medialis, and vastus lateralis), hamstrings (biceps femoris and semitendinosus), and medial gastrocnemius muscles. Electrode placement was performed according to the SENIAM recommendations and the anatomical location of the innervation zones, with an inter-electrode distance of approximately 20 mm (**Figure 2**) (Hermens et al., 2000). To reduce skin impedance, the electrode sites were shaved when necessary and cleaned with alcohol before electrode application. To minimize electrode displacement and movement artifacts during testing, the electrodes were secured using a thin elastic mesh sleeve (Colyer and McGuigan, 2018). Following electrode placement, signal quality was verified by recording brief voluntary muscle contractions. For the FR condition, electrode locations were marked on the skin to ensure consistent repositioning after the intervention (Makaracı et al., 2025). Although electrode locations were marked, their removal and repositioning during FR may have introduced measurement error, as small placement differences can affect normalized sEMG amplitudes.

For sEMG normalization, MVIC measurements were obtained for each muscle group. For the quadriceps muscles, participants performed a maximal isometric knee extension against a dynamometer while seated with the knee positioned at 60° of flexion. For the hamstring muscles, participants performed a maximal isometric knee flexion against manual resistance in the prone position with the knee flexed to 30°. For the medial gastrocnemius muscle, participants performed a maximal heel raise while standing, using light two-finger support to maintain balance. Three trials were completed for each muscle group, and standardized verbal encouragement was provided to ensure maximal effort. The reference MVIC value for each muscle was calculated as the mean of the highest 3-s root mean square (RMS) values obtained across the trials (Burden, 2010). Subsequently, all RMS values recorded during the performance tests were normalized to the corresponding MVIC values and expressed as %MVIC.

Surface electromyographic excitation of the quadriceps, hamstrings, and gastrocnemius muscles was recorded during the vertical and horizontal jump tests. Root mean square (RMS) values were calculated for the push-off phase of both the CMJ and SLHD. In both tests, the push-off phase was defined as the period from maximum knee flexion to take-off. The onset and termination of this phase were determined using the synchronized video recordings integrated into the sEMG system and were subsequently synchronized with the sEMG signals for analysis (Bhanot et al., 2019).

#### 2.3.6 Data processing and analysis

All sEMG signals were recorded at a sampling frequency of 2000 Hz using myoResearch 4.0 software (Noraxon USA Inc., Scottsdale, AZ, USA). The signals were band-pass filtered between 10 and 500 Hz, and root mean square (RMS) values were calculated using a 150-ms moving window. During the CMJ and SLHD tests, the push-off phase was identified from synchronized video recordings, and the corresponding sEMG signals were analyzed. The RMS data were exported to Microsoft Excel (Microsoft Corp., Redmond, WA, USA), where they were normalized to the respective MVIC values for each muscle and expressed as %MVIC. The normalized data were subsequently transferred to IBM SPSS Statistics 27.0 (IBM Corp., Armonk, NY, USA) for statistical analysis.

### 2.4 Statistical analysis

The normality of the data was assessed using the Shapiro–Wilk test, and all variables were found to satisfy the assumption of normal distribution. Accordingly, a two-way repeated-measures analysis of variance (ANOVA) was performed to examine the effects of condition (FR and PR) and time (pre-test, mid-test, and post-test). When significant main effects or interactions were identified, Bonferroni-adjusted pairwise comparisons were conducted. This analytical approach was applied separately to the physical performance outcomes obtained from the SLHD and CMJ tests, as well as to the sEMG excitation of the rectus femoris, vastus lateralis, vastus medialis, biceps femoris, semitendinosus, and medial gastrocnemius muscles recorded during these tests. Effect sizes were calculated as partial eta squared $\left(\eta_{\text{p}}^{2}\right)$ with values greater than 0.01, 0.06, and 0.14 interpreted as small, medium, and large effects, respectively (Cohen, 1988). Statistical significance was set at p< 0.05.

## 3 Results

**Figure 3:**
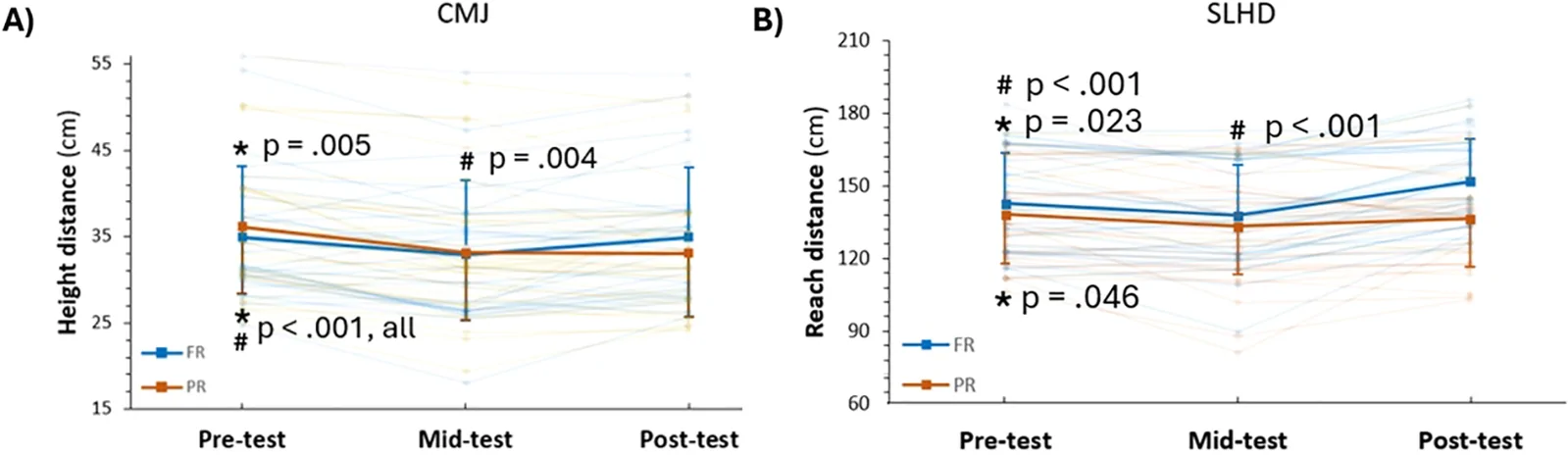
Mean ± standard deviation and pairwise comparisons of **(A)** CMJ and **(B)** SLHD performance in the FR and PR groups. (*p < 0.05, significantly different from the mid-test; #p < 0.05, significantly different from the post-test).

**Figure 4:**
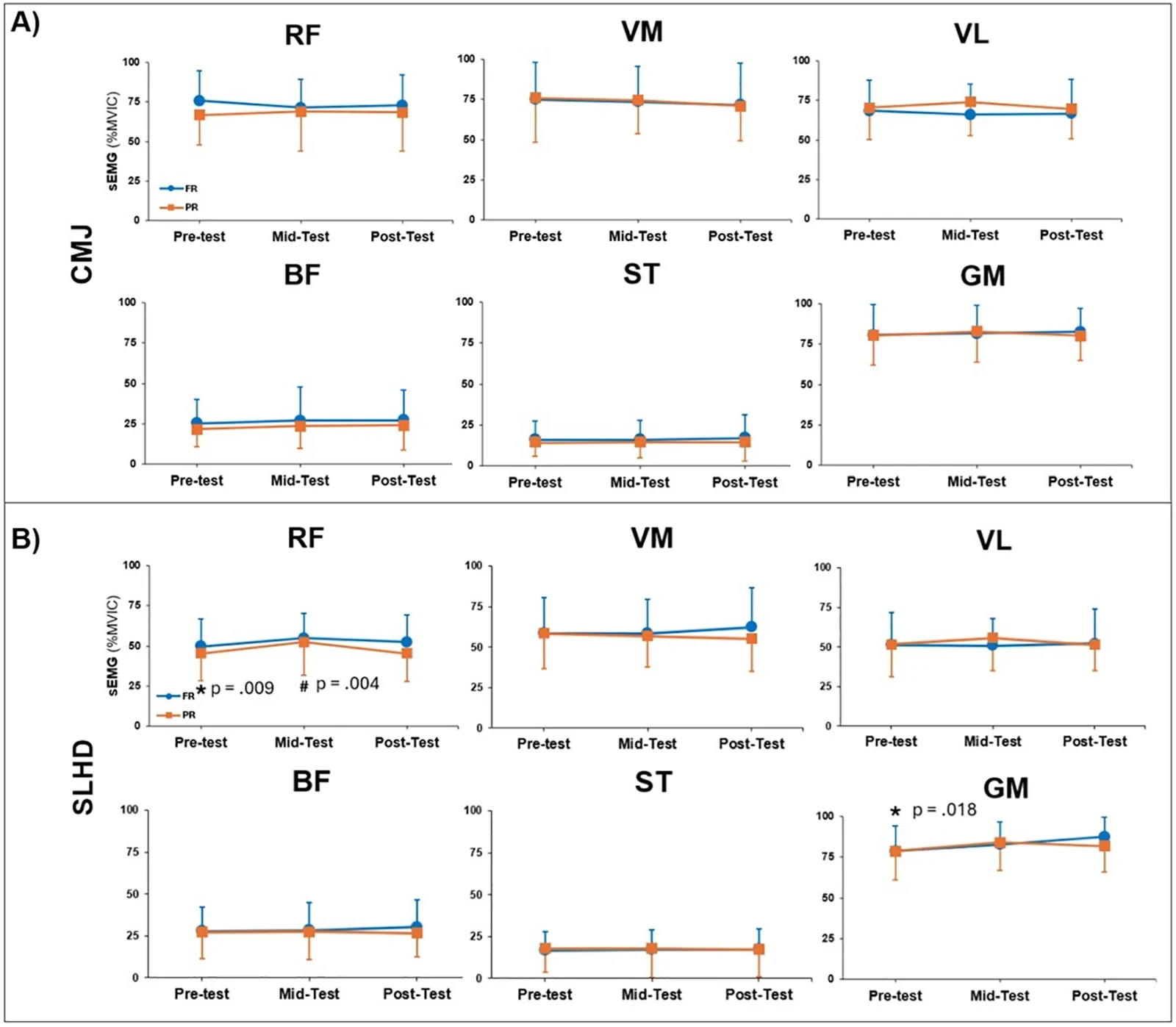
Mean ± standard deviation and pairwise comparisons of **(A)** CMJ and **(B)** SLHD sEMG excitation in the FR and PR groups. (*p < 0.05, significantly different from the mid-test; #p < 0.05, significantly different from the post-test).

The descriptive characteristics of the participants are presented in **Table 1**. The mean values of the physical performance outcomes and the results of the multivariate and pairwise comparisons are presented in **Table 2**, whereas the percentage changes in these variables are illustrated in **Figure 3**. The sEMG excitation recorded during the jump tests is presented as %MVIC in **Figure 4**. Exercise intensity, assessed using the RPE scale, was 18.1 ± 0.9 in the FR condition and 18.2 ± 0.7 in the PR condition (p = .161).

**Table 1:** Descriptive of the participants.

Variable	Mean (SD)	Min	Max
Age (years)	21.6 (1.3)	20.0	24.0
Height (cm)	180.6 (7.2)	165.0	198.0
Weight (kg)	75.3 (10.3)	57.8	101.0
BMI (kg/m^2^)	22.8 (2.3)	18.2	27.5
1 RM (kg)	98.5 (24.0)	60	150
PAL (MET-min/wk.)	1156.1 (808.9)	330.0	4200.0

BMI, body mass index; RM, repetition maximum; PAL, physical activity level.

**Table 2:** Physical performance outcomes of participants: Means, confidence intervals, and ANOVA analysis.

Variable	Time	FR group (*n* = 31)		PR group (*n* = 31)		ANOVA analysis	
		Mean (SD)	[95% CI]	Mean (SD)	[95% CI]	Time x group	Group
CmJ *(cm)*	Pre-test	34.92 (7.26) a	32.26 – 37.59	36.14 (7.71) a,b	33.32 – 38.98	F = 8.253, p < .001*, $\eta_{\text{p}}^{2}=.216$	F = 18.207, p<.001*, $\eta_{\text{p}}^{2}=.378$
	Mid-test	32.89 (7.53) b	30.13 – 35.66	33.11 (7.85)	30.24 – 36.00		
	Post-test	34.94 (7.37)	32.24 – 37.65	33.08 (7.36)	30.39 – 35.79		
SLHD (*cm)*	Pre-test	142.69 (20.74) a,b	135.08 – 150.29	138.13 (19.63) a	130.93 – 145.33	F = 10.346, p<.001*, $\eta_{\text{p}}^{2}=.256$	F = 22.831, p<.001*, $\eta_{\text{p}}^{2}=.432$
	Mid-test	137.70 (20.79) b	130.07 – 145.325	133.32 (23.12)	124.84 – 141.80		
	Post-test	151.84 (17.60)	145.38 – 158.29	136.39 (20.49)	128.88 – 143.90		

ANOVA, analysis of variance; FR, foam rolling; PR, passive recovery; CmJ, countermovement jump; SLHD: single-leg hop for distance; a, significantly different from the mid-test (p< 0.05); b, significantly different from the post-test (p<.05); *statistically significant difference (p<.05).

For the CMJ and SLHD a significant condition × time interactions were observed (p<.05; $\eta_{\text{p}}^{2}=.216\text{ and }.256$, respectively; **Table 2**). Pairwise comparisons revealed a significant decrease in CMJ performance from pre-test to mid-test in the FR condition (Δ = −5.82% [95% CI: −2.59, −8.96]; p = .005), followed by a significant increase from mid-test to post-test (Δ = 6.23% [95% CI: 2.86, 9.95]; p = .004). In the PR condition, significant reductions were observed from pre-test to mid-test (Δ = −8.38% [95% CI: −5.80, −11.00]; p<.001) and from pre-test to post-test (Δ = −8.49% [95% CI: −5.14, −11.49]; p< 0.001). For SLHD performance, pairwise comparisons demonstrated a significant decrease from pre-test to mid-test in the FR condition (Δ = −3.50% [95% CI: −1.24, −5.86]; p = .023), followed by significant increases from mid-test to post-test (Δ = 10.27% [95% CI: 7.48, 13.32]; p<.001) and from pre-test to post-test (Δ = 6.41% [95% CI: 4.14, 8.88]; p<.001). In the PR condition, a significant reduction was observed only from pre-test to mid-test (Δ = −3.48% [95% CI: −1.01, −6.25]; p = .046).

For the normalized sEMG excitation recorded during the CMJ and SLHD tests, no significant condition × time interactions were observed for the other muscles, and all interaction effect sizes were small (p >.05; $\eta_{\text{p}}^{2}=.002-.070$; **Figure 4**; **Supplementary Table 1**). Analysis of the main effect of time revealed significant differences only for the RF and GM muscles during the SLHD test (p<.05; $\eta_{\text{p}}^{2}=.251\text{ and }.187$, respectively), whereas no significant time effects were found for the remaining muscles (p >.05; $\eta_{\text{p}}^{2}=.002-.075$). Pairwise comparisons showed a significant increase in RF muscle excitation from pre-test to mid-test in the PR condition (Δ = 15.96 [95% CI: 6.37, 25.90]; p = .009), followed by a significant decrease from mid-test to post-test (Δ = −13.32 [95% CI: −6.65, −19.73]; p = .004). In the FR condition, a significant increase was observed only in GM muscle excitation from pre-test to post-test (Δ = 11.01 [95% CI: 3.94, 19.38]; p = .018).

## 4 Discussion

The present study investigated the acute effects of FR compared with PR on vertical and horizontal jump performance and lower-extremity muscle excitation following a single session of high-load resistance exercise. The main finding was that FR facilitated the recovery of vertical jump and produced an acute enhancement horizontal jump performance after fatiguing resistance exercise. In contrast, although time-dependent changes were observed in the sEMG excitation of selected muscles, no consistent pattern of neuromuscular excitation was identified across the assessed muscle groups. Overall, FR intervention supported post-exercise jump performance without producing consistent changes in lower-extremity muscle excitation.

Regarding SLHD performance, participants in the FR condition demonstrated a marked increase in performance following the post-exercise decline (Δ = 10.27%; p<.001), whereas only minimal improvement was observed after PR (Δ = 2.30%; p = .456). This finding is consistent with our previous study (Rodoplu et al., 2026b), which reported a comparable improvement in horizontal jump performance after FR following fatiguing resistance exercise (Δ = 8.30%). These findings suggest that FR may support horizontal jump performance following exercise-induced fatigue, with an acute increase in performance observed after the intervention.

Several mechanisms may explain the observed improvement in SLHD performance. Unlike isolated strength assessments, the SLHD is a complex task that depends not only on force production but also on lower-extremity stability, intersegmental coordination, and sensorimotor control (Pilanthananond et al., 2023). Accordingly, successful performance is influenced by proprioceptive function, joint stability, and the mechanical behavior of the muscle–tendon unit in addition to maximal force-generating capacity (Moezy et al., 2008; Kotsifaki et al., 2020; Wang et al., 2022). Previous studies have shown that FR can increase joint range of motion while reducing muscle stiffness (Behara and Jacobson, 2017; Konrad et al., 2021). Such mechanical changes may contribute to force transmission and movement efficiency during unilateral horizontal propulsion; however, these were not directly assessed in the present study (Augustsson et al., 2006). It should be noted that these effects were observed in the non-fatigued muscle, highlighting that FR enhanced performance in the absence of exercise-induced fatigue.

With respect to vertical jump performance, our previous study (Rodoplu et al., 2026b), which also investigated the effects of FR following a fatiguing exercise protocol, found no significant improvement in CMJ performance. Similarly, Laffaye et al. (Sedano and Maroto-Izquierdo, 2025) reported that FR did not attenuate the exercise-induced decline in CMJ performance following a fatiguing exercise protocol. In contrast, Drinkwater et al. (2019) reported improved CMJ performance during the later stages of recovery following eccentric exercise, suggesting that the effects of FR may vary according to the recovery period and exercise model. More recently, Pernigoni et al. (2023) found no additional benefit of FR on CMJ recovery following simulated basketball match-play. In the present study, however, FR produced a significant increase in jump height after the intervention (Δ = 6.20%; p = .004). Furthermore, the systematic review and meta-analysis by Wiewelhove et al. (2019) reported a trivial overall effect of FR on jump performance, highlighting the variability of individual responses across protocols and populations. Taken together, these findings suggest that the effects of FR on horizontal jump performance may be promising, although the available evidence remains limited, whereas its influence on vertical jump performance is less predictable. These differences may be related to the complex biomechanical and neuromuscular demands of the CMJ (Bobbert et al., 1996; Pilanthananond et al., 2023), which may not be consistently influenced by the acute effects of FR.

When the findings from both jump tests are considered together, the most notable observation of the present study is that improvements in performance occurred without a corresponding pattern of consistent sEMG excitation across the lower-extremity muscles. A similar finding was reported by Junker and Stöggl (2019), who observed enhanced horizontal jump performance following FR despite the absence of significant changes in muscle excitation. Collectively, these findings suggest that the acute performance benefits of FR cannot be explained solely by increased motor unit activation. In support of this interpretation, Behm and Wilke (2019) proposed that the effects of FR are likely mediated through multiple interacting mechanisms, including alterations in tissue stiffness, viscoelastic properties, sensory feedback, and other neurophysiological processes, rather than changes in muscle excitation alone. However, these proposed mechanisms should be considered potential explanations, as the physiological basis of the observed performance improvements remains uncertain.

On the other hand, sEMG amplitude represents only one component of neuromuscular function and may not fully account for changes in movement performance. Comparable mechanical outputs can be achieved with similar or even lower levels of muscle excitation through potential differences in intermuscular coordination, motor control, or the mechanical behavior of the muscle–tendon unit (Macgregor et al., 2018). Moreover, the decline in performance following high-load resistance exercise cannot be attributed solely to reductions in muscle excitation. Physiological factors such as impaired excitation–contraction coupling, metabolite accumulation, muscle damage, and peripheral fatigue are also likely to contribute to post-exercise performance decrements (Enoka and Duchateau, 2016). Therefore, the performance changes observed in the present study may involve mechanisms beyond changes in muscle excitation; however, neuromuscular efficiency, movement coordination, and mechanical adaptations of the muscle–tendon unit were not directly assessed in the present study. The dissociation between functional performance and sEMG responses further suggests that the effectiveness of acute recovery interventions should not be evaluated exclusively on the basis of muscle excitation. Instead, the present findings suggest that FR may function primarily as a recovery strategy that may involve mechanisms beyond direct changes in muscle excitation. A methodological consideration is that the sEMG electrodes were temporarily removed to allow the FR intervention and subsequently repositioned before the post-test measurements. To minimize placement variability, the electrode locations were marked on the skin before removal and repositioned according to these markings while adhering to the SENIAM recommendations. Nevertheless, slight differences in electrode repositioning may have introduced minor variability in the recorded sEMG amplitudes and should be considered when interpreting the present findings. This interpretation is consistent with previous studies suggesting that FR and roller massage may influence functional performance, movement quality, and neuromuscular efficiency through mechanisms extending beyond changes in muscle activation (Bradbury-Squires et al., 2015; Behm and Wilke, 2019; Rodoplu et al., 2026b; Rodoplu et al., 2026a).

From a practical standpoint, the present findings suggest that FR may represent a useful recovery strategy for supporting post-exercise performance following high-load resistance exercise. This may be particularly relevant during periods of intensified training, schedules involving limited recovery time between training sessions or competitions, and activities requiring repeated explosive efforts, where maintaining both vertical and horizontal jump performance is of functional importance. Nevertheless, the present results indicate that FR should be regarded as an intervention that facilitates post-exercise recovery and performance maintenance rather than as a method that directly enhances athletic performance.

Several limitations of this study should be acknowledged. First, only young healthy male participants were included, which may limit the generalizability of the findings to female athletes or other populations (e.g., older individuals or athletes with different training backgrounds). Second, the study examined only the acute effects of FR, whereas the long-term effects of different application durations, intensities, and treatment protocols remain unclear. In addition, sEMG recordings were limited to selected lower-extremity muscles. Furthermore, electrode removal and repositioning during FR may have introduced measurement error, as small differences in electrode placement can affect normalized sEMG amplitudes; therefore, the sEMG-derived muscle excitation findings should be interpreted with caution. The relatively large number of dependent variables should also be considered when interpreting the findings because of the potential for Type I error. Future studies should include a broader range of muscles, such as the gluteal muscles, soleus, and tibialis anterior, together with complementary assessments including muscle stiffness, proprioceptive function, force-platform variables, and markers of muscle damage to provide a more comprehensive understanding of the mechanisms through which FR influences performance and recovery.

## 5 Conclusion

FR performed following a single session of high-load resistance exercise facilitated the restoration of vertical jump performance and produced an acute enhancement in horizontal jump performance compared with PR. In contrast, the normalized sEMG findings did not demonstrate a consistent increase in lower-extremity muscle excitation following FR. These results suggest that the acute performance benefits of FR may involve mechanisms beyond changes in muscle excitation, including potential changes in proprioceptive feedback, the viscoelastic properties of the muscle–tendon unit, muscle stiffness, and neuromuscular efficiency. Nevertheless, these mechanisms remain speculative because they were not directly assessed in the present study. From a practical perspective, FR may represent an easily applicable recovery strategy that may support post-exercise jump performance following high-load resistance exercise. Future studies should investigate different FR protocols using more comprehensive neuromuscular and biomechanical assessments to further clarify the mechanisms underlying these performance adaptations.

## Data Availability

The original contributions presented in the study are included in the article/**Supplementary Material**. Further inquiries can be directed to the corresponding author.
