# Supplementary material for: Acute effects of foam rolling on jump performance and neuromuscular responses following resistance exercise
Source: Front Physiol. 2026 Sep 9;17:1945152. doi: 10.3389/fphys.2026.1945152 (PMC13597403; doi:10.3389/fphys.2026.1945152)
Supplement: Supplementary file 1 [file Table1.docx]

Supplementary Material

# 1 Supplementary Data

**Table S1.** Participants’ EMG Activities Normalized to MVC: Means, Confidence Intervals, and ANOVA Analysis

| **Variable** | **Time** | **FR** | |  | **PR** | |  | **Intervention X Time** | | |  | **Time** | | |
| --- | --- | --- | --- | --- | --- | --- | --- | --- | --- | --- | --- | --- | --- | --- |
|  |  | **Mean (SD)** | **[95% CI]** |  | **Mean (SD)** | **[95% CI]** |  | **F _(2, 60)_** | ***p*** | $\boldsymbol{\eta}_{\boldsymbol{p}}^{\boldsymbol{2}}$ |  | **F _(2, 60)_** | ***p*** | $\boldsymbol{\eta}_{\boldsymbol{p}}^{\boldsymbol{2}}$ |
| **CmJ** | Pre-test | 75.79 (18.75) | 68.91 – 82.66 |  | 66.90 (19.02) | 59.92 – 73.88 |  |  |  |  |  |  |  |  |
| *RF* | Mid-test | 71.48 (17.84) | 64.94 – 78.03 |  | 68.96 (25.10) | 59.76 – 78.17 |  | 1.355 | .266 | .043 |  | .217 | .806 | .007 |
|  | Post-test | 72.98 (19.18) | 65.94 – 80.01 |  | 68.53 (24.44) | 59.57 – 77.50 |  |  |  |  |  |  |  |  |
|  | Pre-test | 75.03 (23.09) | 66.56 – 83.50 |  | 76.06 (24.70) | 67.01 – 85.12 |  |  |  |  |  |  |  |  |
| *VM* | Mid-test | 73.68 (22.04) | 65.60 – 81.77 |  | 74.50 (20.81) | 66.87 – 82.13 |  | .089 | .915 | .003 |  | 2.416 | .098 | .075 |
|  | Post-test | 71.57 (26.26) | 61.94 – 81.21 |  | 70.95 (21.43) | 63.09 – 78.81 |  |  |  |  |  |  |  |  |
|  | Pre-test | 68.45 (19.25) | 61.38 – 75.51 |  | 70.41 (20.23) | 62.99 – 77.83 |  |  |  |  |  |  |  |  |
| *VLO* | Mid-test | 66.13 (19.30) | 59.06 – 73.21 |  | 74.08 (21.44) | 66.21 – 81.94 |  | 2.179 | .122 | .068 |  | .698 | .502 | .023 |
|  | Post-test | 66.90 (21.62) | 58.97 – 74.83 |  | 69.64 (18.71) | 62.78 – 76.50 |  |  |  |  |  |  |  |  |
|  | Pre-test | 25.35 (14.66) | 19.98 – 30.73 |  | 21.61 (10.88) | 17.62 – 25.60 |  |  |  |  |  |  |  |  |
| *BF* | Mid-test | 27.13 (20.62) | 19.56 – 34.69 |  | 23.61 (13.69) | 18.59 – 28.63 |  | .047 | .954 | .002 |  | 2.024 | .150 | .063 |
|  | Post-test | 27.26 (18.69) | 20.41 – 34.12 |  | 24.14 (15.41) | 18.49 – 29.79 |  |  |  |  |  |  |  |  |
|  | Pre-test | 16.04 (11.43) | 11.84 – 20.23 |  | 14.66 (8.75) | 11.45 – 17.87 |  |  |  |  |  |  |  |  |
| *ST* | Mid-test | 16.18 (11.69) | 11.89 – 20.47 |  | 14.69 (9.79) | 11.10 – 18.28 |  | .175 | .840 | .006 |  | .240 | .787 | .008 |
|  | Post-test | 17.07 (14.37) | 11.80 – 22.34 |  | 14.75 (11.55) | 10.52 – 18.99 |  |  |  |  |  |  |  |  |
|  | Pre-test | 80.58 (19.10) | 73.58 – 87.59 |  | 80.33 (18.11) | 73.69 – 86.98 |  |  |  |  |  |  |  |  |
| *GM* | Mid-test | 81.96 (17.59) | 75.51 – 88.42 |  | 82.86 (18.82) | 75.96 – 89.76 |  | .431 | .652 | .014 |  | .652 | .525 | .021 |
|  | Post-test | 82.55 (14.75) | 77.14 – 87.96 |  | 80.16 (15.30) | 74.55 – 85.77 |  |  |  |  |  |  |  |  |
| **SLHD** | Pre-test | 49.56 (17.54) | 43.13 – 56.00 |  | 45.18 (16.67) **a** | 39.07 – 51.30 |  |  |  |  |  |  |  |  |
| *RF* | Mid-test | 54.84 (15.25) | 49.24 – 60.43 |  | 52.39 (20.59) **b** | 44.84 – 59.95 |  | 1.237 | .297 | .040 |  | 10.034 | **<.001** | .251 |
|  | Post-test | 52.32 (17.07) | 46.06 – 58.58 |  | 45.41 (17.52) | 38.99 – 51.84 |  |  |  |  |  |  |  |  |
|  | Pre-test | 58.31 (21.94) | 50.27 – 66.36 |  | 58.38 (21.68) | 50.43 – 66.34 |  |  |  |  |  |  |  |  |
| *VM* | Mid-test | 58.47 (20.98) | 50.78 – 66.17 |  | 56.73 (19.26) | 49.66 – 63.79 |  | 2.264 | .113 | .070 |  | .161 | .852 | .005 |
|  | Post-test | 62.18 (24.56) | 53.17 – 71.19 |  | 55.13 (20.13) | 47.74 – 62.51 |  |  |  |  |  |  |  |  |
|  | Pre-test | 51.35 (20.52) | 43.82 – 58.88 |  | 51.67 (20.57) | 44.13 – 59.22 |  |  |  |  |  |  |  |  |
| *VLO* | Mid-test | 50.83 (16.89) | 44.64 – 57.03 |  | 55.69 (20.76) | 48.08 – 63.30 |  | 1.751 | .182 | .055 |  | .685 | .508 | .022 |
|  | Post-test | 52.07 (21.74) | 44.10 – 60.04 |  | 51.20 (16.35) | 45.20 – 57.20 |  |  |  |  |  |  |  |  |
|  | Pre-test | 27.84 (14.32) | 22.58 – 33.09 |  | 27.30 (15.65) | 21.56 – 33.04 |  |  |  |  |  |  |  |  |
| *BF* | Mid-test | 28.17 (16.58) | 22.09 – 34.25 |  | 27.51 (16.65) | 21.40 – 33.62 |  | .912 | .407 | .030 |  | .259 | .773 | .009 |
|  | Post-test | 30.11 (16.33) | 24.12 – 36.10 |  | 26.71 (14.38) | 21.44 – 31.99 |  |  |  |  |  |  |  |  |
|  | Pre-test | 16.67 (11.32) | 12.52 – 20.82 |  | 17.85 (14.28) | 12.61 – 23.09 |  |  |  |  |  |  |  |  |
| *ST* | Mid-test | 17.29 (11.58) | 13.04 – 21.54 |  | 17.75 (17.04) | 11.50 – 24.00 |  | .120 | .887 | .004 |  | .052 | .949 | .002 |
|  | Post-test | 17.28 (12.21) | 12.80 – 21.76 |  | 17.54 (17.56) | 11.10 – 23.98 |  |  |  |  |  |  |  |  |
|  | Pre-test | 78.84 (15.55) **b** | 73.13 – 84.54 |  | 78.73 (17.67) | 72.25 – 85.21 |  |  |  |  |  |  |  |  |
| *GM* | Mid-test | 82.86 (13.77) | 77.81 – 87.91 |  | 84.03 (16.95) | 77.81 – 90.25 |  | 2.274 | .123 | .070 |  | 6.908 | **.004** | .187 |
|  | Post-test | 87.51 (14.20) | 82.30 – 92.72 |  | 81.90 (16.07) | 76.01 – 87.80 |  |  |  |  |  |  |  |  |
| ANOVA: analysis of variance; FR: foam rolling; PR: passive recovery; CmJ: countermovement jump; SLHD: single‑leg hop for distance; **a**: significantly different from the mid-test (p < 0.05); **b:** significantly different from the post-test (p < .05); ***:** statistically significant difference (p < .05). | | | | | | | | | | | | | | |
|  | | | | | | | | | | | | | | |
|  | | | | | | | | | | | | | | |
